# The effect of perturbation-based balance training on balance control and fear of falling in older adults: a single-blind randomised controlled trial

**DOI:** 10.1186/s12877-023-03988-x

**Published:** 2023-05-17

**Authors:** Marissa Gerards, Rik Marcellis, Rachel Senden, Martijn Poeze, Rob de Bie, Kenneth Meijer, Antoine Lenssen

**Affiliations:** 1 Department of Physiotherapy, Maastricht University Medical Center (MUMC+), Maastricht, The Netherlands; 2 Department of Epidemiology, Maastricht University, Maastricht, The Netherlands; 3 Department of Nutrition and Movement Sciences, Maastricht University, Maastricht, The Netherlands; 4 Department of Surgery, division of Trauma Surgery, MUMC+, Maastricht, The Netherlands; 5 Care and Public Health Institute (CAPHRI), Maastricht University, Maastricht, The Netherlands; 6 School of Nutrition and Translational Research in Metabolism (NUTRIM), Maastricht University, Maastricht, The Netherlands

**Keywords:** Accidental Falls, Aging, Balance, Perturbation, Prevention, Older adults

## Abstract

**Background:**

Perturbation-based balance training (PBT) is an emerging intervention shown to improve balance recovery responses and reduce falls in everyday life in older adults. However, perturbation interventions were heterogeneous in nature and need improvement. This study aims to investigate the effects of a PBT protocol that was designed to address previously identified challenges of PBT, in addition to usual care, on balance control and fear of falling in older adults at increased risk of falling.

**Methods:**

Community-dwelling older adults (age ≥ 65 years) who visited the hospital outpatient clinic due to a fall incident were included. Participants received PBT in addition to usual care (referral to a physiotherapist) versus usual care alone. PBT consisted of three 30-minute sessions in three weeks. Unilateral treadmill belt accelerations and decelerations and platform perturbations (shifts and tilts) were applied during standing and walking on the Computer Assisted Rehabilitation Environment (CAREN, Motek Medical BV). This dual-belt treadmill embedded in a motion platform with 6 degrees of freedom is surrounded by a 180° screen on which virtual reality environments are projected. Duration and contents of the training were standardised, while training progression was individualised. Fear of falling (FES-I) and balance control (Mini-BESTest) were assessed at baseline and one week post-intervention. Primary analysis compared changes in outcome measures between groups using Mann-Whitney U tests.

**Results:**

Eighty-two participants were included (PBT group n = 39), with a median age of 73 years (IQR 8 years). Median Mini-BESTest scores did not clinically relevantly improve and were not significantly different between groups post-intervention (p = 0.87). FES-I scores did not change in either group.

**Conclusions:**

Participation in a PBT program including multiple perturbation types and directions did not lead to different effects than usual care on clinical measures of balance control or fear of falling in community-dwelling older adults with a recent history of falls. More research is needed to explore how to modulate PBT training dose, and which clinical outcomes are most suitable to measure training effects on balance control.

**Trial registration:**

Nederlands Trial Register NL7680. Registered 17-04-2019 – retrospectively registered. https://www.trialregister.nl/trial/7680.

**Supplementary Information:**

The online version contains supplementary material available at 10.1186/s12877-023-03988-x.

## Introduction

Falls annually affect one in three adults over 65 years [1, 2]. In 2019, approximately 109.000 Dutch adults over 65 years visited the emergency department due to a fall incident, resulting in over 1 billion euros of direct medical costs [3]. Falls are a leading cause of injuries and hospitalization among older adults and can have many physical and psychological adverse consequences [4, 5]. The risk of falling increases with age and can increase even further in the presence of additional risk factors. An important and modifiable risk factor for falls is balance control [6, 7]. Having experienced a fall incident is another prognostic factor, which greatly increases the risk of sustaining future falls (odds ratio (OR) 2.8 for all fallers, and 3.5 for recurrent fallers) [6, 8, 9]. Consequently, older adults who have fallen are also more likely to develop fear of falling, and to experience a further decline in balance control after their fall incident [10, 11]. Since fear of falling and impaired balance control both increase the risk for falls, this illustrates how a single fall incident can cause older adults to end up in a negative cycle [10]. Given that the number of older adults is currently increasing rapidly, the burden of falls will continue to increase. Therefore, it is essential to develop interventions that make older adults more resistant to fall incidents.

Balance training is reported to effectively improve balance control and reduce fall rates in older adults by approximately 24% [12, 13]. Nonetheless, there are some drawbacks to conventional balance training interventions. Firstly, it should be understood that ‘balance control’ is an umbrella concept that can be subdivided into multiple motor skills [14], each of which is vital to perform activities of daily living. As described by Berg et al., balance control can be divided into three general categories: maintaining a position (static balance control), adjusting voluntary or anticipated movements (proactive balance control), and reacting to external or unexpected balance perturbations (reactive balance control) [15]. Conventional balance training programs do not typically address reactive balance control but focus mostly on proactive balance control [16]. However, approximately 60% of falls in older adults result from an unexpected external perturbation during walking, such as a slip or trip [17]. As the recovery actions needed to prevent such falls rely mostly on reactive balance control, the task-specificity of conventional balance training may be limited [18, 19]. Additionally, conventional balance training requires a relatively high number of training sessions to be effective, and retention of training effects is hard to accomplish [20–22]. As such, research to optimize balance training interventions for older adults continues.

In recent years, perturbation-based balance training (PBT) has gained increasing interest as an intervention which is more task-specific to the recovery actions needed to prevent falls from unexpected balance perturbations. PBT aims to improve reactive balance by repeatedly exposing participants to destabilizing perturbations in a safe and controlled environment. Results from two meta-analyses indicate that PBT can significantly reduce daily-life fall rates by 46% and 52%, respectively [23, 24]. Studies have also found beneficial effects of PBT on the number of laboratory-induced falls [25–27], various measures of balance recovery (e.g. margins of stability after perturbation or time to stabilization of center of pressure) [25, 27–32], and balance control (e.g. limits of stability, five-step test, and Berg Balance Scale (BBS)) [33–36]. Additionally, PBT may have the potential to be effective with a low training dose (one to four training sessions), which could make PBT a more efficient alternative to other interventions [37–40].

While current studies report promising results of PBT, they are heterogeneous in terms of training parameters, study populations, and outcomes. Further study is required to help develop effective training protocols that are tolerable and acceptable to the target population. To build upon the literature, this study will address some previous identified challenges of PBT. Firstly, studies have suggested that PBT training effects may be limited to the specific condition that was trained [39, 41], or only partly generalizable to different conditions [18, 42]. This is a factor that should be addressed to ensure a beneficial impact on everyday life, where falls can happen in all kinds of conditions. A potential strategy that has been proposed to improve the generalization of adaptations to PBT is to include a variety of training conditions (for example multiple perturbation types and directions) [43]. Secondly, other challenges include the physical tolerability and acceptability of PBT for older adults. Not all older adults may initially be able to tolerate the required training dose of PBT, which may lead to anxiety or inability to physically cope with the perturbations [40]. Anxiety during training is a factor that was found to limit acceptability and increase drop-out rates, and thus limit the effectiveness of PBT [32]. A proposed method to improve both physical tolerability as well as acceptability is to progressively increase training intensity (e.g. perturbation magnitude or unexpectedness) in a manner that is tailored to the individual [26, 40, 43].

In this study, we evaluate the additive effects of a three-session PBT protocol [44] that was designed to address these previously identified challenges of PBT, in addition to usual care, on balance control and fear of falling in older adults with an increased risk of falling based on a recent fall incident.

## Methods

### Study design and participants

A detailed description of the full study protocol was previously published [44]. In this single-blinded randomised controlled trial (RCT) PBT was offered to community-dwelling older adults (≥ 65 years). Older adults were eligible to participate in the study if they had experienced a fall in the previous three months, had therefore visited the hospital outpatient clinic, and were able to walk without a walking aid for ≥ 15 min. Exclusion criteria were diagnosis of any disease or disorder that may affect the safety of training (e.g. osteoporosis, recent fracture or severe contusion of the lower extremities, back or shoulders, or severe cardiopulmonary disease), falls caused by actions of third parties or during exercise activities, falls due to syncope, and use of medication known to increase falls risk [45]. Potential participants were also excluded if they were unable to follow instructions due to cognitive problems, unable to provide written informed consent or to communicate in Dutch. The study intervention and measurements were conducted between March 2019 and August 2021 at the physiotherapy department of the Maastricht University Medical Center, The Netherlands. Study outcomes were measured at baseline and one week post-intervention. Ethics approval was obtained from the Medical Ethics Committee azM/UM (METC18-049, Maastricht University and Maastricht University Medical Center), and the study was conducted in accordance with the declaration of Helsinki. All participants provided written informed consent.

### Interventions

Participants were randomised to receive PBT as an add-on to usual care versus usual care alone.

#### Usual care

Usual care consisted of physiotherapy referral by the medical doctor if indicated. As in usual clinical practice, it was ultimately up to the patient to decide whether or not to visit the physiotherapist. Both the content and duration of the physiotherapy treatment could vary based on the needs of each individual patient, and were decided by the medical specialist and physiotherapist in consultation with the patient. During study visits, the outcome assessor routinely monitored if and how often each participant had visited their physiotherapist, and what components (i.e., strength training, mobility exercises, balance exercises) were included in the physiotherapy treatment.

#### Perturbation-based balance training

Participants referred to the experimental group received usual care as described above, and three 30-minute sessions of PBT additionally. The PBT sessions were given once a week for three consecutive weeks using the Computer Assisted Rehabilitation Environment (CAREN, MOTEK Medical BV). The CAREN is a dual-belt treadmill embedded in a motion platform with 6 degrees of freedom. The treadmill and motion platform can both provide unexpected balance perturbations, such as unilateral treadmill belt accelerations or decelerations, and platform translations and rotations in various directions. This is combined with a 180-degree screen on which a virtual reality environment is projected to make training more immersive. Participants wear a safety harness during training sessions, preventing the knees from hitting the ground in case of an unsuccessful balance recovery.

### Training procedures

The first training session on the CAREN started with a familiarization procedure, where the participant could get used to the system by walking on the treadmill in the virtual environment. Participants reported a numeric rating scale (NRS) score for how comfortable they felt when walking on the CAREN before and during familiarization (0; very uncomfortable to 10; fully comfortable). If a score of 7 or higher was reached, the familiarization procedure was deemed complete. This was expected to occur within 6–7 min [46].

After this, each participants’ comfortable walking speed was determined using a ramp protocol. Subjects started walking at 0.5 m/s and speed was gradually increased until the subject said ‘stop’ when their comfortable walking speed was reached. The participant then walked unperturbed at this speed for approximately one minute to check if any adjustments needed to be made. Due to the procedures for familiarization and determining the comfortable walking speed, the total duration of the first training session could be up to 40 min.

Consecutive sessions started with a warm-up during which the participant walked unperturbed on the treadmill at their comfortable speed (determined during the first session) for approximately 3 min and got readjusted to the system. Each training session consisted of three parts: gait adaptability, static and dynamic reactive balance control (of which details are given below). During training, participants were regularly asked to rate how challenging it was to maintain their balance on an NRS score (0–10; 0 = not challenging at all, 10 = unable to maintain balance). To ensure that the training was challenging but acceptable and tolerable for each participant, the aim was to train at a difficulty level between 6 and 9 (challenging to very challenging) on the NRS. Training progression was based on these NRS scores, and each participant’s ability to manage the perturbations. Training difficulty was progressed by increasing the perturbation intensity and walking speed. During the second and final training sessions, cognitive and motor dual tasks (e.g., counting backwards in steps of 7, hitting targets in the virtual environment) could also be added to increase training difficulty. Participants were aware that there would be ‘various challenges to their balance’ during the training, and were instructed to recover their balance and to try to continue walking. Participants were naïve to the timing and the order of perturbation types that would be applied. Training adherence (number of training sessions attended and completed, reasons for missed training sessions) was monitored throughout the study, and participants were encouraged to reschedule any missed training sessions.

#### Gait adaptability

Participants walked in a virtual environment of a path through a forest, with various slopes and turns. Both the incline/decline of the slopes and the sharpness of the turns had a standardised starting level of 20% (out of 100%), which was then progressed in steps of 5–15%. Each 5% increase in difficulty level means an increase in the incline, decline and rotation of 0.5 degrees.

#### Static reactive balance

Participants stood on the CAREN while the platform and treadmill belts provided sudden perturbations. The platform could shift (move in the horizontal plane) or tilt (move into a sloping position) to anterior, posterior, left and right. The treadmill belt could unilaterally accelerate from standstill. In the second and third parts of the first training session, training difficulty started at the lowest difficulty level for each participant and could be progressed individually over 7 difficulty levels. Details on perturbation characteristics for each difficulty level were published previously [44].

#### Dynamic reactive balance

Participants walked on the treadmill at their comfortable walking speed, while the above-mentioned platform and treadmill perturbations were applied. The treadmill perturbations consisted of unilaterally accelerating or decelerating the treadmill belt for short periods of time (0.2–0.7 s).

### Measurements

Study outcomes were measured at baseline (T0) and after 4 weeks, which was one week after the final training session for the PBT group (T1), by an outcome assessor who was blinded to treatment allocation. Demographic data (age in years, sex, body height (cm) and weight (kg)) was collected at baseline, including retrospective falls incidence over the previous 12 months. A fall was defined as ‘an event which results in a person coming to rest inadvertently on the ground or floor or lower level’ [47]. An adapted version of the ‘falls history questionnaire’ as presented in the book *‘Falls in older people: risk factors and strategies for prevention’* was used [48]. This questionnaire records if a fall has occurred in the previous 12 months, where it has happened, what the perceived cause was, and if and what kind of injuries were sustained. The outcome assessor filled in this questionnaire together with the subject to make sure that the recorded data was as comprehensive and clear as possible. Adherence to training in the PBT group was defined as attendance to the training session and completion of at least two out of three training parts.

#### Balance control

The main outcome in this study was balance control, assessed on the Mini-BESTest. The Mini-BESTest is a comprehensive measurement tool to assess balance in community-dwelling older adults [49]. The test is divided into four categories: anticipatory balance control (0–6 points), reactive balance control (0–6 points), sensory orientation (0–6 points) and dynamic gait (0–10 points). There are a total of 14 tasks which are scored on a three-point scale (0–2), with a total score ranging between 0 and 28 points. A higher score corresponds with better balance control. The Mini-BESTest has good reliability and validity, and a significantly smaller ceiling effect in community-dwelling older adults compared to the BBS [50, 51]. The minimal detectable change (MDC) at the 95% confidence level is 3–4 points [52–54]. In a study by Magnani et al., age-dependent cut-off values were determined, where scoring below a certain value was associated with an increased risk of falling in the next 6 months (area under the ROC curve 0.68–0.79); <25 points for people 60 to 69 year of age, < 23 points for 70 to 79 years, < 22 points for 80 to 89 years and < 17 points for 90 years and older [55].

#### Fear of falling

Fear of falling was measured with the Falls Efficacy Scale International (FES-I, Dutch version). This version of the falls efficacy scale is a 16-item questionnaire developed to determine if a person has confidence in their ability to perform a range of daily activities without falling. It has been adapted to be more suited to older adults, including a range of activities from very basic to more complex [56]. The questionnaire will be filled in by the subject with the help of the outcome assessor. Sixteen items are scored on a four-point (1–4) scale, with a maximum score of 64 points. A higher score corresponds with a greater fear of falling. Cut-off points of 16–22 points for ‘low concern’ and 23–64 points for ‘high concern’ about falling have been described [57]. The Dutch version of the FES-I has good reliability and validity and discriminative power in older adults [56–59].

### Sample size and randomisation

Sample size was calculated in G*power 3.1.9.2 and was based on the primary outcome of this study, difference between group means on balance measured with the Mini-BESTest post-intervention. The effect size *d* (0.61) was calculated based on values from an article with a similar study population and an intervention aimed at improving dynamic balance control [52]. We assumed that changes from baseline to post-intervention would be in the favour of the PBT group. Sample size was based on a one-tailed independent samples t-test, with an α of 0.05, power (1-β) of 0.80 and allocation ratio of 1. This resulted in a required sample size of n = 72. Accounting for an expected loss to follow-up of 10%, the final sample size is n = 80. Sample size was estimated conservatively, making no assumptions about the correlation between predictors (group allocation and baseline score) added into the model and the outcome variable.

Participants were randomised using a 1:1 ratio stratified block randomisation (block sizes 2 and 4). Randomisation was stratified based on sex (male/female) and number of falls during the previous year (1 versus 2 or more falls). The randomisation sequence was generated by an independent researcher using an online random number generator. Participants were enrolled and allocated to their groups by the main researcher. Allocation was concealed by using sequentially numbered, sealed opaque envelopes. The allocation sequence list was kept in a locked drawer, which could only be accessed by the principal investigator.

### Statistical analysis

Descriptive statistics are presented as means and standard deviations (SD) or as medians and interquartile ranges (IQR), depending on the normality of the data. Categorical data is presented as frequencies (n) or percentages (%). Data was analysed on an intention-to-treat basis (missing data imputed using single stochastic regression imputation). Mann-Whitney U tests were used to determine if there was a significant between-group difference in Mini-BESTest or FES-I score change between baseline and follow-up (ΔMini-BESTest or ΔFES-I, respectively). To compare proportions or percentages between groups, the chi-square test was used. A multiple regression analysis was performed to explore potential confounding variables in the association between group and ΔMini-BESTest. Variables (age, sex, previous falls, FES-I at baseline, if the participant visited a physiotherapist and if their treatment included gait/balance training between baseline and post-intervention) were added (enter method) to the model, and variables that resulted in a ≥ 10% change in the regression coefficient of the main determinant were eligible for inclusion in the model. The variable contributing the most (≥ 10%) was included in the model first, and this process was repeated for each following variable until there were no more potential confounding factors [60]. In all analyses, statistical significance was set at p < 0.05. Data was analysed using the Statistical Package for the Social Sciences version 23.0 (SPSS, Chicago, Ill., USA).

## Results

**Fig. 1:**
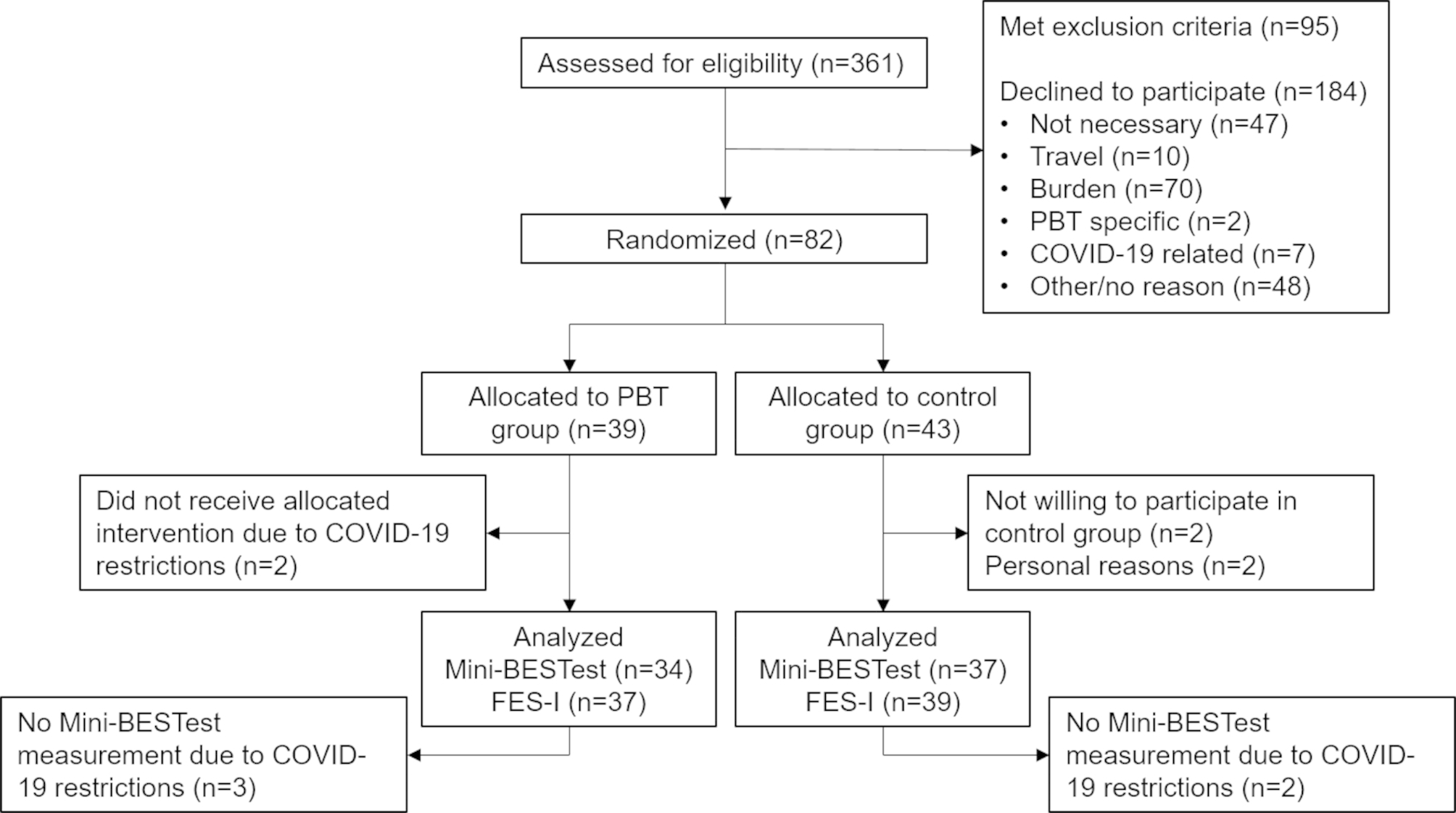
CONSORT flow-diagram of participants. Overview of participant flow from inclusion to the final measurements, including reasons for non-inclusion or drop-outs. PBT = perturbation-based balance training. Mini-BESTest = Mini Balance Evaluation Systems Test. FES-I = Falls Efficacy Scale International. Not necessary = participant did not think they needed an intervention. Travel = participant did not want to participate due to travel issues. Burden = participant considered the time investment too high (often in combination with other treatment appointments). PBT-specific = two participants did not wish to participate for reasons related to the PBT training, specifically the safety harness. COVID-19 related = participants did not wish to participate due to the COVID-19 pandemic.

Between March 27th, 2019 and July 8th, 2021, 82 participants were included, of which 39 were randomly assigned to the PBT group (8 men, 31 women) and 43 to the control group (9 men, 34 women). The median age of the participants was 73 years (IQR 8 years). Some 49 participants experienced 1 fall during the previous year, 19 participants had fallen twice and 14 participants had fallen 3 or more times. No significant between-group differences in demographics and baseline characteristics of the participants were found (see Table 1*)*. Six participants withdrew from the study; 2 (PBT group) due to restrictions related to the COVID-19 pandemic, 2 due to personal reasons (control group) and 2 no longer wanted to participate when they were randomised to the control group. Mini-BESTest values of five more participants (2 control and 3 PBT) are missing due to restrictions related to the COVID-19 pandemic. Figure 1 gives an overview of the flow of participants from eligibility assessment to each stage of the study.

### Training characteristics

Adherence to the training was 93.7% over all training sessions (104 out of 111 total scheduled sessions), of which 100 sessions were fully completed. Of the 37 participants who started PBT training, 31 (83.8%) attended all three training sessions. Six participants missed one or two training sessions, due to co-morbidities (n = 3), scheduling issues (n = 2) or feeling that their balance was too good for the training (n = 1). One participant reported a training-related adverse event of experiencing knee pain after making a misstep during the training session, which resolved without intervention within two days after the training session. Perturbation difficulty level was progressed individually, aiming to train at an NRS score of 6–9, representing the subjective experience of how challenging the perturbations were for each participant on a scale of 0 (not challenging) to 10 (unable to maintain balance). Progressing to higher perturbation difficulty levels (levels ranging from 1 (smallest perturbations) to 7 (largest perturbations)) based on this score, enabled the therapists to individualize training progression for each participant. By the end of the first session, participants on average reached perturbation difficulty levels 4 (range 2–6) and 2 (range 1–5) for standing and walking perturbations respectively (7 being the highest difficulty level). By the end of the third training session, participants on average reached level 6 (range 3–7) for standing perturbations and level 4 (range 2–7) for walking perturbations.

**Table 1:** Participant characteristics

	Control (n = 43)	PBT (n = 39)	p-value
Age (years)	73 (8)	73 (10)	0.77
Sex (male/female)	9/34	8/31	0.96
Height (cm)	164.0 (10.0)	161.0 (11.6)	0.50
Weight (kg)	69.7 (18.5)	71.1 (19.0)	0.73
BMI (kg/m_2_)	26.3 (4.2)	27.0 (5.0)	0.71
Falls in previous 12 months (1, 2, ≥ 3 (n))	1 (25), 2 (12), ≥ 3 (6)	1 (24), 2 (7), ≥ 3 (8)	0.90
Physiotherapy T0-T1 (yes/no)	19/24	20/19	0.52
Gait/Balance training T0-T1 (yes/no)	4/39	4/35	0.88

Data is presented as median (interquartile range) or frequencies. Physiotherapy T0-T1 and Gait/balance training T0-T1; if participants received any physiotherapy sessions between T0 and T1, and if this training included gait and/or balance training. BMI = Body Mass Index

### Assumptions

All assumptions for statistical tests were met. An analysis of standard residuals showed that the data contained one outlier on the Mini-BESTest at T1. As this score accurately reflected the performance measured for this participant, this value was not removed from the analysis.

### Outcomes

**Fig. 2:**
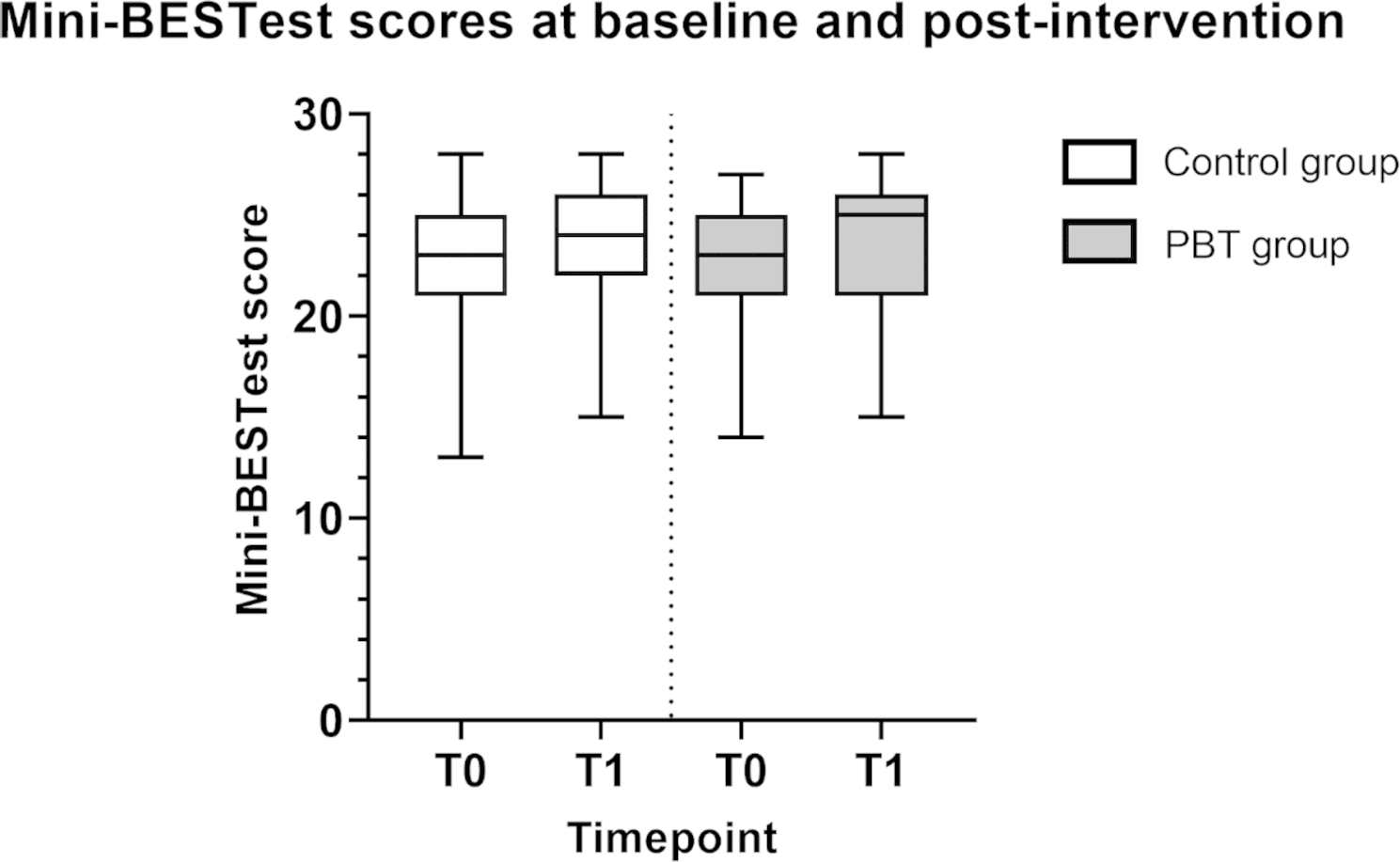
Mini-BESTest scores at baseline and post-intervention in the control- and PBT groups. Mini-BESTest: Mini Balance Evaluation Systems test. T0: baseline, T1: post-intervention

Table 2 shows outcomes at baseline (T0) and post-intervention (T1) for both groups. Mini-BESTest scores at T0 and T1 are presented in Fig. 2. Baseline Mini-BESTest scores were 23 (4) points in both groups, baseline FES-I scores were 20 (8) and 20 (7) points for the control group and PBT group, respectively (median (IQR)). Median Mini-BESTest scores increased in both groups, however these change scores were not significantly different between groups (p = 0.87). No significant between-group difference in FES-I change scores was found (p = 0.85).

Multiple regression analysis was performed to explore and correct for potential interacting or confounding variables (age, sex, previous falls, FES-I at baseline, physiotherapy T0-T1 and Gait/Balance training T0-T1) in the association between group and ΔMini-BESTest, and between group and ΔFES-I. This analysis revealed that there was a significant interaction effect of age and receiving physiotherapy on the association between group and change in Mini-BESTest. Age also acted as a confounder in the relationship between group and change in FES-I. Adding these variables to the models did not result in a significant association in either model. Full regression analysis results and tables are reported in Additional file 1.

**Table 2:** Outcome measures at baseline and post-intervention

	Control			PBT			
	T0	T1	change	T0	T1	change	*p*
Mini-BESTest (0–28)	23 (4)	24 (4)	1 (3)	23 (4)	25 (5)	1 (3)	0.87
Anticipatory balance control (0–6)	5 (1)	5 (2)	0 (1)	4 (1)	5 (2)	0 (1)	0.45
Reactive balance control (0–6)	4 (2)	5 (2)	1 (2)	5 (2)	5 (2)	0 (2)	0.28
Sensory orientation (0–6)	6 (0)	6 (0)	0 (0)	6 (1)	6 (0)	0 (0)	0.91
Dynamic gait (0–10)	8 (1)	9 (2)	1 (1)	8 (1)	9 (2)	0 (1)	0.55
Increased fall risk, n(%)	23 (50.0)	12 (26.1)	11 (23.9)	21 (56.8)	15 (40.5)	6 (16.3)	0.23
FES-I (16–64)	20 (8)	20 (7)	0 (3)	20 (7)	19 (7)	0 (3)	0.85

Outcome measures (range of possible scores) for the control- and PBT-group at T0 (baseline) and T1 (post-intervention). Change = difference between pre- and post- outcome values. Mini-BESTest = Mini Balance Evaluation Systems Test. FES-I = Falls Efficacy Score International. Increased fall risk = the percentage of participants at increased risk for falling based on their Mini-BESTest score. Data is presented as median (IQR) or n (percentage)

## Discussion

This study evaluated the effects of a PBT protocol in addition to usual care on balance control and fear of falling in older adults with a recent history of falls. We hypothesised that the PBT group would show greater improvements compared to the control group. While median Mini-BESTest scores increased slightly in both groups, these changes were below the threshold for minimal detectable change and were not significantly different between groups. Median falls efficacy scores decreased by one point in the PBT group but not in the control group, and no significant between-group differences were found. Explorative secondary analysis revealed interactive effects of age and receiving physiotherapy on the association between group and ΔMini-BESTest, while age acted as a confounding variable on the association between group and ΔFES-I. Correction for confounding variables strengthened both associations but did not lead to a significant association with group in either.

The findings of this study do not support our hypothesis that balance control measured with the Mini-BESTest would improve significantly in the PBT group compared to the control group. Similar conclusions were drawn by recent studies comparing the effects of PBT to general balance training or multimodal physiotherapy on clinical measures of balance control (BBS, Timed Up and Go, and Dynamic Gait Index) in healthy community-dwelling older adults [61, 62]. As all participants in the present study had recently experienced one or more fall incidents, we expected that this would be reflected in their baseline values for balance control [11]. However, Mini-BESTest scores at baseline were already high, and based on these scores only approximately half (50 − 57%) of the participants were considered at increased risk for falls [55]. To our knowledge, the only studies that found significantly greater improvements in clinical measures of balance control after PBT compared to other interventions included populations with neurological conditions such as Parkinson’s disease, with notably lower scores at baseline [34, 35]. The results of this study, in line with recent literature in healthy community-dwelling older adults, did not show a significant additional effect of PBT to usual care on clinical measures of balance control in community-dwelling older adults who visit a hospital outpatient clinic due to a fall. The high Mini-BESTest scores at baseline could indicate that this outcome measure may not be sufficiently sensitive to changes in balance control in relatively healthy community-dwelling older adults. Thus, while studies have indicated that the Mini-BESTest can be useful to evaluate effects of PBT in populations with more impaired balance control, a potential avenue for future research may be to explore which clinical measures of balance control are suitable to evaluate the effects of PBT in this population.

Transfer of training adaptations to other tasks or contexts is an important challenge of PBT. Studies have shown that transfer of training adaptations can be achieved, but so far only to different conditions for a similar task. For example, studies have found transfer of PBT training adaptations after slip-perturbations on a low-friction moveable platform to a slippery floor [63], or from a treadmill to an overground surface [42], and interlimb transfer of training adaptations in the same perturbation type and context [64]. Therefore, the training protocol in the current study was designed with the aim to facilitate transfer of training adaptations by including a broader range of perturbation types and directions. However, despite each participant being able to progress to higher perturbation levels during training, this did not transfer to significant changes in overall balance control or balance recovery from lean-and-release perturbations in the reactive balance sub score measured with the Mini-BESTest. These results imply that the application of multiple perturbation types and directions in a three-session PBT protocol may not be sufficient to generate meaningful transfer to clinical measures of balance control in community-dwelling older adults with a recent history of falls. However, as development and optimization of PBT interventions are still emerging research topics, the implications of these findings for transfer of PBT training adaptations should be interpreted cautiously. For one, it should be considered which outcome measures are most suitable to measure training effects on balance control. While a clinical measure of balance control such as the Mini-BESTest may be more feasible to use in clinical practice, more subtle changes after balance training may be more accurately measured by instrumented measures (such as postural sway or gait parameters), as was demonstrated in a recent study by Hasegawa et al. [65]. More research is needed to determine which outcome measures are sufficiently sensitive to changes in balance control after training, and how they correlate with meaningful changes in balance control for everyday life.

Additionally, while studies have shown promising beneficial effects of PBT with a single or few training sessions [26, 37, 38], there are no strong guidelines on how to modulate training load to attain an optimal effect [66]. While the training dose in this study was based on a previous literature review [67], it should be considered that a higher training dose might be required to elicit adaptations on balance control that can be measured with a clinical balance test such as the Mini-BESTest. More research is needed to elucidate the training dose-response relationship of PBT, also considering the effects of factors such as perturbation intensity (high versus low or progressive intensity), transferability and retention of training adaptations.

No significant between-group differences were found in change of fear of falling measured with the FES-I. Median values at baseline were classified as ‘low concern’ based on previously determined cut-off values, and stayed the same for the control group and decreased by one point for the PBT group [57]. These findings are in line with a previous study that found no significant group-by-time interaction effects on fear of falling after PBT [68].

The results of this study show high training adherence rates, one training-related adverse event, and that increasing training difficulty was possible for each participant, confirming the feasibility of this PBT protocol for participants. However, including participants in the study proved challenging. Figure 1 shows that approximately half of the potentially eligible older adults that were approached, declined to participate. This is comparable to the median inclusion rates of 48.5% in falls prevention interventions for older adults, reported by Nyman et al. [69]. The most prevalent reasons older adults mentioned were that despite their recent fall(s), they did not see themselves as needing falls prevention or balance training, or that the burden of five study visits (including three training sessions) was too high. These reasons are also comparable to common barriers to participation in falls prevention, while only 2 potential participants indicated that they did not wish to participate for reasons specifically related to the PBT [70].

### Limitations

This study was not without limitations. Firstly, restrictions related to the COVID-19 pandemic meant that some adjustments had to be made to the treatment protocol. The inclusion criterion of having experienced a fall in the previous 3 months was broadened to the previous 6 months to increase inclusion rates and reach the required sample size after a period of lockdown measures. In retrospect, this was the case for a similar number of participants in the PBT and control groups, and if this was of significant influence this would be expected to be visible from the baseline participant characteristics. The same restrictions also meant that some of the follow-up FES-I measurements had to be done over the phone, this was the case for 2 and 3 participants in the PBT and control group, respectively. This data was collected by the same outcome assessor as the baseline measurements, and did not lead to any issues. Second, while common in intervention studies, participants and therapists in the intervention group were not blinded to group allocation. However, therapists providing usual care, outcome assessors and data analysers were blinded. Additionally, the measurements were performed by a blinded outcome assessor that encouraged all participants to give their best effort. A final limitation of this study is that no direct measures of balance control in response to perturbations were applied during training. While this was a conscious choice to enable participants to focus on their training experience, it also means that direct training effects cannot be analysed from this study.

## Conclusions

Participation in a PBT program that includes multiple perturbation types and directions did not lead to significantly different effects than usual care on balance control measured with the Mini-BESTest in this population of community-dwelling older adults with a recent history of falls. Fear of falling measured with the FES-I did not change in either group. More research is needed to elucidate the training dose-response relationship of PBT, and how to modulate PBT training load to attain optimal and transferrable training adaptations. Additionally, further study is needed to explore which clinical outcomes are suitable to measure PBT training effects on balance control in community-dwelling older adults.

## Electronic supplementary material

Below is the link to the electronic supplementary material.

Additional file 1: Regression analysis results

## Data Availability

The datasets used and analysed during the current study are available from the corresponding author on reasonable request.

## Abbreviations

PBT: Perturbation-based balance training
Mini-BESTest: Mini balance evaluation systems test
FES-I: Falls efficacy scale international
IQR: Interquartile range
OR: Odds ratio
BBS: Berg balance scale
RCT: Randomised controlled trial
CAREN: Computer assisted rehabilitation environment
NRS: Numeric rating scale
T0: Measurement time, baseline
T1: Measurement time, post-intervention
SD: Standard deviation
ΔMini-BESTest: Change in Mini-BESTest
ΔFES-I: Change in FES-I
