# Supplementary material for: The effect of perturbation-based balance training on balance control and fear of falling in older adults: a single-blind randomised controlled trial
Source: BMC Geriatr. 2023 May 17;23:305. doi: 10.1186/s12877-023-03988-x (PMC10191085; doi:10.1186/s12877-023-03988-x)
Supplement: Supplementary file 1 — Additional file 1: Regression analysis results [file 12877_2023_3988_MOESM1_ESM.docx]

# Additional file 1: Regression analysis results

### Assumptions

All assumptions for statistical tests were met. An analysis of standard residuals showed that the data contained one outlier (subject 75, standardized residual -3,769). As this was a plausible outcome value, this participant was not removed from the analysis.

Multiple regression analysis was performed to explore and correct for potential interacting or confounding variables (age, sex, previous falls, FES-I at baseline, physiotherapy T0-T1 and Gait/Balance training T0-T1) in the association between group and ΔMini-BESTest, and between group and ΔFES-I. *Table S1* shows the results of univariate linear regression, with no significant association between group and ΔMini-BESTest. Multiple linear regression analysis revealed a significant interaction effect of age (participants with above median age showed smaller improvements when in the intervention group, p = 0.004), and receiving physical therapy (participants with physical therapy improved less in both groups, this effect was counteracted if participants received physical therapy and PBT, p=0.047). Addition of these factors to the model did not result in a significant association between group and ΔMini-BESTest. No confounding variables were identified.

**Table S1. *Linear regression analysis – the association between group and ΔMini-BESTest.***

|  |  |  |  | *95% Confidence Interval for B* | |
| --- | --- | --- | --- | --- | --- |
|  | *B* | *Std. Error* | *p-value* | *Lower bound* | *Upper Bound* |
| *Constant* | *1.000* | *0.339* | *0.004* | *0.325* | *1.675* |
| *Group* | *-0.308* | *0.492* | *0.53* | *-1.287* | *0.672* |

The association between group and ΔFES-I was explored in a similar approach, with the same potential interacting or confounding variables (baseline FES-I was exchanged for baseline Mini-BESTest). *Table S2* shows the results of univariate linear regression, with no significant association between group and ΔFES-I. No significant interaction effects were found. The addition of age resulted in a 11,1% change in the regression coefficient of the main determinant (group) and was added to the model (*Table S3*). The remaining variables were then added to the model corrected for age, but none resulted in a ≥10% change in the regression coefficient thus no more variables were added to the model. The results show that correcting for age strengthened the association between group and ΔFES-I, but this did not lead to a significant association.

**Table S2. L*inear regression analysis – the association between group and ΔFES-I.***

|  |  |  |  | *95% Confidence Interval for B* | |
| --- | --- | --- | --- | --- | --- |
|  | *B* | *Std. Error* | *p-value* | *Lower bound* | *Upper Bound* |
| *Constant* | *-0.326* | *0.526* | *0.54* | *-1.372* | *0.720* |
| *Group* | *-0.315* | *0.762* | *0.68* | *-1.832* | *1.201* |

**Table S3. *Multiple linear regression analysis – the association between group and ΔFES-I, corrected for age.***

|  |  |  |  | *95% Confidence Interval for B* | |
| --- | --- | --- | --- | --- | --- |
|  | *B* | *Std. Error* | *p-value* | *Lower bound* | *Upper Bound* |
| *Constant* | *3.336* | *5.278* | *0.53* | *-7.169* | *13.842* |
| *Group* | *-0.350* | *0.766* | *0.46* | *-1.875* | *1.175* |
| *Age* | *-0.050* | *0.072* | *0.49* | *-0.193* | *0.093* |
